# A deadly prescription: combination of methotrexate and trimethoprim-sulfamethoxazole

**DOI:** 10.1080/20009666.2018.1466598

**Published:** 2018-06-12

**Authors:** Mohsin Hamid, Bilal Lashari, Irfan Ahsan, Ida Micaily, Usman Sarwar, Joseph Crocetti

**Affiliations:** a Department of Internal Medicine, Abington Hospital–Jefferson Health, Abington, PA, USA; b Pulmonary and Critical Care, Abington Hospital–Jefferson Health, Abington, PA, USA

**Keywords:** Methotrexate, trimethoprim-sulfamethoxazole, deadly drug interaction, folinic acid rescue, pancytopenia

## Abstract

Methotrexate (MTX) is a chemotherapeutic synthetic(s) phase cell cycle inhibitor, and its role has evolved as an immunological agent in autoimmune diseases like rheumatoid arthritis, psoriasis, and systemic lupus erythematosus, etc. Trimethoprim-sulfamethoxazole (TS) is one of the most widely prescribed antibiotics commonly used for urinary tract infections, exacerbations of chronic bronchitis, traveler’s diarrhea, and pneumocystis pneumonia. Both MTX and TS can have significantly overlapping side effects involving dermatologic, renal, and hematological systems, and the combination of these can be deadly. Our case is about the combination of MTX and TS that leads to mucocutaneous ulceration, leukopenia, and renal insufficiency. The purpose of this case is to increase awareness of potentially significant toxicity from the combination of MTX with TS.

**Abbreviations:** MTX: methotrexate; TS: trimethoprim-sulfamethoxazole; ED: emergency department; IV: intravenous; GI: gastrointestinal; NSAIDs: nonsteroidal anti-inflammatory drugs.

## 1. Introduction

Methotrexate (MTX) is a chemotherapeutic synthetic(s) phase cell cycle-specific inhibitor that preferentially targets actively proliferating tissues. It irreversibly binds and inhibits dihydrofolate reductase enzyme and impairs the ability of the DNA to replicate. Being the first chemotherapeutic agent shown to have a response in cancer treatment, it changed the world of chemotherapy as we know it and led to the development of many standard cancer treatments used today [1-3]. The role of MTX has evolved as an immunological agent in autoimmune diseases such as rheumatoid arthritis, psoriasis, and systemic lupus erythematosus, etc. The most common adverse effects include gastrointestinal (GI) upset, stomatitis, rash, neurological symptoms (headache, fatigue, agitation), mild transaminitis, and macrocytosis [4]. As an immunological agent, lower doses are used; however, toxicities can still occur due to interactions with other medications or impairments in renal and hepatic metabolism.

Trimethoprim-sulfamethoxazole (TS) is one of the most widely prescribed antibiotic medications and works as a bacteriostatic antimicrobial by inhibiting sequential enzymes in the folic acid pathway. Sulfamethoxazole blocks the formation of dihydrofolic acid from its precursors, and trimethoprim blocks the reduction of dihydrofolate into tetrahydrofolate. It is commonly used for conditions such as urinary tract infections, exacerbations of chronic bronchitis, traveler’s diarrhea, and pneumocystis pneumonia. Its potential toxicities include nephrotoxicity, hyperkalemia, pancytopenia, neutropenia, skin rash, and Steven Johnson’s syndrome many of which overlap with the toxicity of MTX [5]. We are presenting this case to increase awareness of the potentially significant toxicity of MTX due to its interaction TS.

## 2. Case presentation

A 68-year-old female with a history of rheumatoid arthritis on 10 mg MTX weekly presented to the emergency department (ED) with weakness, lethargy, and decreased oral intake. Two weeks prior to presentation, her primary care physician (PCP) prescribed a 2-week course of TS for presumed bacterial skin infection under her left breast. One week after starting therapy, she noticed painful oral sores and flu-like symptoms. In the ED, she was hypotensive to 90/60 mm Hg but responded to intravenous (IV) fluids. Vital signs were otherwise stable. The pertinent physical exam included severe oral mucositis, lip ulceration, and an erythematous rash under her left breast. Laboratory data revealed creatinine: 2.39 mg/dl (baseline 0.7 mg/dL), potassium: 5.3 mEq/L, chloride: 97 mEq/L, leukocyte count: 3.2 K/uL, absolute neutrophil count: 2.8 K/uL, hemoglobin: 12.3 mg/dL, and platelet count: 230 K/uL. TS was held as the symptoms of mucocutaneous ulceration, leukopenia, and renal insufficiency were initially attributed to possible TS toxicity and supportive care with IV fluids and oral care was given. Her oral ulcers remained unresolved; leukocyte count continued to drop reaching a nadir of 1.9 K/uL and absolute neutrophil count dipped to 0.7 K/uL over the course of next 2 days. Renal function, however, improved with IV hydration. After consultation with hematology, a careful review of patient’s medications list and drug interactions were done, and her symptoms were attributed to MTX–TS interaction leading to MTX toxicity rather than toxicity of TS itself. MTX levels were sent that came back high at 0.5 µmol/L. Leucovorin rescue was given at the dose of 10 mg/m^2^ every 6 h for a total of four doses with one dose of Filgrastim 480 mcg which resulted in the improvement of the leukocyte count, breast rash, and mouth ulcers within 24 h. The patient was discharged on day 6 of hospitalization and did well post discharge.

## 3. Discussion

The first serious interaction between TS and MTX was reported in 1986 by Thomas [6]. Since then, numerous case reports and observational studies have been published but the combination is still prescribed and in many instances has led to fatal consequences [7,8].

After oral administration, MTX is absorbed quickly, the kidneys excrete 80–90% and liver enzymes metabolize the rest. A positive correlation has been noticed between MTX clearance and creatinine clearance and hence abnormal renal function can precipitate MTX toxicity. After filtration by glomeruli, it undergoes both secretion and reabsorption and competes with other drugs during this process. It accumulates in third spaces such as pleura and peritoneum and is eliminated slowly from these spaces as compared to plasma, and the dosage needs to be readjusted in these situations. The primary metabolite is 90% bound to plasma proteins and therefore can have extensive drug interactions. Most commonly co-prescribed drugs with MTX are steroids, aspirin (ASA), and nonsteroidal anti-inflammatory drugs (NSAIDs). Steroids have been shown to be safe with MTX. NSAIDs and high dose ASA decrease the renal excretion of MTX and increase its plasma concentration. However, a systematic review found that concurrent use of most NSAIDs with MTX is safe, yet, anti-inflammatory doses of ASA should not be used with MTX [9]. Folate has been shown to reduce the toxicity of MTX especially GI and liver toxicity without affecting the efficacy of MTX through unknown mechanisms [10,11].

Multiple case reports and other observational studies have noted severe interaction between MTX and TS [6–8,9,12–19]. When used together, TS can increase MTX’s toxicity. Although the exact mechanism is unknown, synergistic folate antagonism, competitive tubular secretion, and displacement from albumin binding site might explain it [8,20–22]. One study pointed out an increase of about 60% of free MTX concentration in blood when used concurrently with TS [15]. The most commonly reported toxicity of MTX due to its interaction with TS is pancytopenia, acute megaloblastic anemia, stomatitis, and nephrotoxicity [19]. Other case reports have also shown rare side effects such as toxic epidermal necrolysis [23]. Care should be taken not to confuse toxicity of MTX with TS as TS has similar side effect profile namely nephrotoxicity, pancytopenia, and Steven Johnson’s syndrome. In the case of possible MTX toxicity, serum levels should be checked and if found to be high, aggressive IV hydration, urinary alkalization, and folinic acid rescue should be initiated. Therapy should be continued until the MTX dose levels have dropped to 0.05–0.1 µmol/L or less.

It has however been noticed that prophylactic dose of TS does not have any significant interaction when used concurrently with MTX and can be co-prescribed safely.

Medical health professional needs to be aware of the differences between the toxicity of MTX and TS and their potential interaction so that they will not falsely attribute the toxicity of MTX to TS.

## 4. Conclusion

With the available current literature, it is prudent to say that the combination of MTX and TS should be avoided, as it can lead to significant morbidity and possibly mortality. To prevent this combination, primary care doctors need to be educated about this deadly combination as the prescription of TS in outpatient clinics usually bypasses the pharmacy-based safety checkpoints. The patients taking MTX should be instructed about the potential side effects and interactions so that prompt identification and discontinuation can be done to prevent serious harm.
